# DO DEPRESSIVE SYMPTOMS MEDIATE SEGREGATION’S IMPACT ON COGNITIVE AGING IN OLDER MEXICAN AMERICANS?

**DOI:** 10.1093/geroni/igad104.3718

**Published:** 2023-12-21

**Authors:** Emily Morris, Jeremy Grant, Alexandra Clark, Darlingtina Esiaka, Kristine Ajrouch, Wassim Tarraf

**Affiliations:** University of Michigan, Ann Arbor, Michigan, United States; University of Florida, Gainseville, Florida, United States; University of Texas at Austin, Austin, Texas, United States; University of Kentucky, Lexington, Kentucky, United States; Eastern Michigan University, Ypsilanti, Michigan, United States; Wayne State University, Detroit, Michigan, United States

## Abstract

Segregation in the United States has important implications for cognitive aging among Mexican Americans as a social determinant of health. Because segregation is systemically engrained, identifying more immediately modifiable mechanisms, such as depressive symptoms, that may underly segregation-cognition associations could point to intervention targets to reduce cognitive aging disparities. We used path mediation modeling to test whether depressive symptoms mediated (indirect effect) the segregation-cognition associations. We used H-EPESE data on older Mexican Americans from the first (n=3,050) wave and returning at wave 5 (n=1,167) combined with Census data from the National Neighborhood Data Archive, which provided indicators for county level segregation (isolation, interaction, dissimilarity). Cognition was operationalized as baseline MMSE, MMSE at wave 5, and change between baseline and wave 5. Depressive symptoms were measured by the CESD at baseline. Covariates included age, sex, and years of education. The sample was 58% female with an average age of 73.6 (SD=6.2) and an average 5-years education (SD=3.9). Higher dissimilarity (i.e., less exposure to non-Hispanic Whites) was associated with better baseline (B=.078, SE=.031, p=.012) and wave 5 cognition (B=.130, SE=.037, p<.001), independent of depressive symptoms. More depressive symptoms were associated with worse baseline and wave 5 cognition, independent of segregation indices (CESD B values range from -.063 to -.142, ps <.05). Depressive symptoms did not mediate segregation-cognition associations. Results suggest that there may be an enclave effect that is protective for both depressive symptoms and cognition in older Mexican Americans. Future research should examine additional biopsychosocial mechanisms underlying segregation-cognition associations.

